# Cell Dissociation from Butterfly Pupal Wing Tissues for Single-Cell RNA Sequencing

**DOI:** 10.3390/mps3040072

**Published:** 2020-10-28

**Authors:** Anupama Prakash, Antónia Monteiro

**Affiliations:** 1 Department of Biological Sciences, National University of Singapore, 14 Science Drive 4, Singapore 117543, Singapore; 2 Yale-NUS College, 10 College Avenue West, Singapore 138609, Singapore

**Keywords:** *Bicyclus anynana*, wing dissociation, single cells, single cell sequencing, FACS

## Abstract

Butterflies are well known for their beautiful wings and have been great systems to understand the ecology, evolution, genetics, and development of patterning and coloration. These color patterns are mosaics on the wing created by the tiling of individual units called scales, which develop from single cells. Traditionally, bulk RNA sequencing (RNA-seq) has been used extensively to identify the loci involved in wing color development and pattern formation. RNA-seq provides an averaged gene expression landscape of the entire wing tissue or of small dissected wing regions under consideration. However, to understand the gene expression patterns of the units of color, which are the scales, and to identify different scale cell types within a wing that produce different colors and scale structures, it is necessary to study single cells. This has recently been facilitated by the advent of single-cell sequencing. Here, we provide a detailed protocol for the dissociation of cells from *Bicyclus anynana* pupal wings to obtain a viable single-cell suspension for downstream single-cell sequencing. We outline our experimental design and the use of fluorescence-activated cell sorting (FACS) to obtain putative scale-building and socket cells based on size. Finally, we discuss some of the current challenges of this technique in studying single-cell scale development and suggest future avenues to address these challenges.

## 1. Introduction

**Figure 1:**
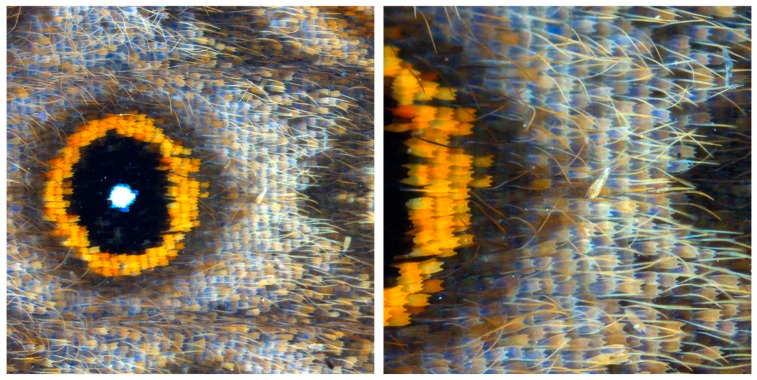
An adult *Bicyclus anynana* ventral hindwing showing the tiled arrangement of scales. Each scale is a projection of a single cell and produces only one color. The overall pattern is created by the mosaic arrangement of differently colored scales. Here, hair-like scales are also visible.

Members of the order Lepidoptera have been great model systems to answer questions from the fields of ecology to developmental biology and genetics. They are characterized by the presence of cellular projections called scales on their wings and bodies. In butterflies specifically, scales are often brightly colored and arranged in intricate mosaic patterns on the wing that serve various ecological functions [1,2,3,4,5]. Color is produced either by chemical pigments deposited in the scale, by nanostructures on the scale that create color by pigment-free, physical processes such as interference, or a combination of the two [6,7]. Typically, scales are arranged on the wing membrane in neat rows with alternating larger cover scales and smaller underlying ground scales (Figure 1) [8], attaching to the wing membrane via sockets. Each scale and its corresponding socket are the products of a single scale-building cell and a socket cell. Scales first appear on developing wing discs as flattened sacks, becoming increasingly sculpted over the course of development [9,10]. The basic scale structure that remains once the cell that builds it dies, just prior to adult eclosion, consists of a highly patterned scaffold made up of the polymer chitin, cuticular proteins, waxes, and pigments. A typical scale consists of a lower lamina thin film connected to an intricately- patterned upper lamina via pillars called trabeculae. Modifications to various parts of this structure produce immense diversity in scale shape and organization, often paralleled by modifications in color [11,12,13]. How single cells control and produce this diversity of nanopatterned structures still remains a mystery. 

For decades, biologists have been trying to understand the developmental genetics and molecular mechanisms of color production and patterning across different butterfly species. Traditionally, bulk RNA sequencing is used to compare transcriptome profiles of differently-colored wing regions to identify the loci important for color development [14,15,16,17]. However, these techniques average gene expression from millions of cells that make up the tissue, thereby masking underlying cellular heterogeneity such as the distinction between cover and ground scale populations and dorsal and ventral cell populations that are often differently colored. To be able to characterize this cellular heterogeneity it is important to study gene expression at the single-cell level.

With the advent of single-cell RNA sequencing [18,19,20,21], it is now possible to compare gene expression profiles across thousands of individual cells, allowing for the characterization of different cell types within a wing tissue. The major steps in a single-cell sequencing experiment involve the preparation of a single-cell suspension, isolation of single cells, capture and amplification of the minute amounts of mRNA inside cells, preparation of barcoded libraries, and sequencing and analysis of the data [21,22]. The first and most important step of a single-cell sequencing experiment is therefore the preparation of a viable, single-cell suspension from tissues of interest. 

For this protocol, the tissue of interest is a butterfly pupal wing, where scale cells are determined from undifferentiated wing epithelial cells in the early pupal stages. Based on studies in the butterflies *Junonia coenia* [23] and *Vanessa cardui* [10], sensory organ precursor cells (SOP cells), which are the precursors to the scale and socket cells, are determined about 12 h after pupation (AP) (~6–7% of pupal development, where the average developmental time from pupation to eclosion in these species is about 8 and 7 days, respectively). These cells are arranged in neat rows that run along the anterior–posterior axis and can be clearly distinguished from the underlying epithelial cells because of their larger size. The SOP then undergoes two rounds of cell division. Following the first round of cell division at around 15 h AP, one of the daughter cells dies. By 24 h AP (~12–14% of development), the other daughter cell has started dividing to produce the scale and socket cells. Scales begin to project from the scale cells around 36 h AP (~21% of development for *V. cardui*). In this protocol, we describe the technique we used in the lab to prepare a viable single-cell suspension from *Bicyclus anynana* 24 h pupal wings (~14% of development, where average pupal development time is about 7 days), detailing all the steps necessary to obtain a viable cell suspension of required concentration for downstream single-cell RNA sequencing. The methods described here can be adapted to different butterfly species and wings from larval stages up to 48 h AP (~28% of pupal development in *B. anynana* and *V. cardui*), providing a resource to successfully implement a single-cell sequencing experiment using butterfly wings, a technique still in its infant stages within the lepidopteran community. 

## 2. Experimental Design

**Figure 2:**
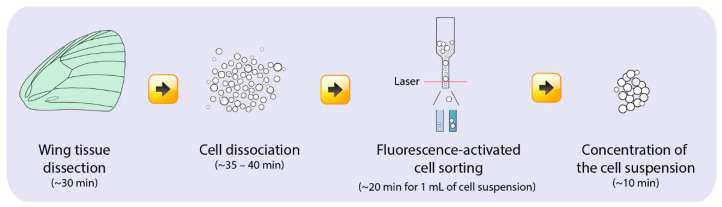
Workflow for the preparation of a viable single-cell suspension of putative scale-building and socket cells from *Bicyclus anyana* pupal wing tissues and the time needed for each step.

The main experimental stages for the preparation of a single-cell scale suspension include: (1) dissection of the wing tissues, (2) cell dissociation from the wing tissues, (3) fluorescence-activated cell sorting (FACS), and (4) concentration of the single-cell suspension (Figure 2). This is followed by single cell sorting into individual emulsion droplets, library preparation, and single-cell sequencing which was done through an independent service (Genome Institute of Singapore). We used 10x Genomics for the single cell partitioning and library preparation step. It is important to note that the choice of technique for downstream cell partitioning and library preparation depends on the depth and breadth of cell profiling required [21,22,24]. All the stages in this protocol have to be done on the same day to ensure the use of live cells. 

### 2.1. Dissection of the Wing Tissues

Developing wing tissues can be dissected at different time points and the single-cell transcriptomes will apply to wing cell populations at that time point exclusively. In our experiment, we dissected *B. anynana* pupal wings at 24 h AP (14% of pupal development) and hence this protocol is optimized for 24 h pupal wings. We have noticed that 24–48 h AP (14–28% of development) are good time points for cell dissociation and believe that larval wings are also amenable to this process. However, in *B. anynana*, older pupal wing tissues, beyond 3 days AP (~42% of pupal development), are harder to dissociate and often lead to many dead cells with developing scales broken from their corresponding cell. The break occurs in the thin pedicel region that connects the body of the cell to the main cell projection that makes up the actual scale. At these later time points a protocol focusing on nuclear transcript sequencing may be more appropriate, but it is still unclear to us whether broken cells, with the scale projection part separated from the cell nucleus part, are viable.

### 2.2. Cell Dissociation from the Wing Tissues 

A single-cell suspension is prepared by dissociating cells from the wing tissues using proteolytic enzymes.

### 2.3. Fluorescence-Activated Cell Sorting (FACS)

A butterfly wing disc is a complex tissue consisting of many cell types including muscle cells, tracheal cells, hemolymph cells, pheromone synthesis and secretory cells, neurons, and epithelial cells, with the latter being the largest fraction. Cells that develop scales are specified as SOP cells early in pupal development and undergo two rounds of cell division that ultimately lead to the formation of a scale and socket cell [10,23]. These precursor cells, scale-building cells, and socket cells are larger in size and polyploid as compared to the underlying smaller, diploid epithelial cells [10,23,25]. Fluorescence-activated cell sorting is used to sort the population of single cells from the wing disc to obtain putative scale-building and socket cells based on their size. This is an optional step but without it, it is likely that a user will obtain data primarily from the underlying epithelial cells because they form the largest fraction.

### 2.4. Concentration of the Single-Cell Suspension

This step is done to concentrate the cell suspension after FACS to obtain the necessary concentrations required for the single-cell library preparation.

### 2.5. Materials

- Microcentrifuge tubes 1.5 mL (Eppendorf, Germany; Cat. no.: T9661-500EA).
- Pipette tips—1 mL, 200 μL, 10 μL (Axygen, CA, USA; Cat. nos.: 14-222-690, 14-222-812, and 14-222-737).
- Silicone for dissection plates (Dragon Skin 30 Mould Making Silicone Rubber; Cat. no.: 0751635278417).
- Petri dishes (Sigma-Aldrich, Singapore; Cat. No.: P5981-100EA).
- 41 µm Nylon Net (Merck Millipore, Germany; Cat. no.: NY4102500 100 filters, 25 mm).
- Countess cell counting chamber slides (Invitrogen by Thermo Fisher Scientific, Waltham, MA, USA; Cat. no.: C10228).
- DNA LoBind Tubes 1.5 mL (Eppendorf, Germany; Cat. no.: 022431021).
- Flow-Check Pro Fluorophores for FACS (Beckman Coulter, Brea, CA, USA; Cat. no.: A63493) (provided by the NUS Medicine Flow Cytometry Lab).

### 2.6. Equipment

- Class II Biological Safety cabinet (LabCard, Lenexa, KS, USA).
- 37 °C incubator (Yihder Co. Ltd.; Model no.: LM-420D).
- Pipettes: 200–1000 μL, 20–200 μL, 0.5–10 μL (Eppendorf Research or Research Plus; Eppendorf, Germany).
- Refrigerated centrifuge (Eppendorf 5810 R, Eppendorf; Hettich MIKRO 220R, Hettich ZENTRIFUGEN, Frankenberg, Germany).
- Countess Automated cell counter (Invitrogen by Thermo Fisher Scientific, Waltham, MA, USA).
- Beckman Coulter Moflo Astrios (FACS Cell Sorter, Beckman Coulter, Brea, CA, USA).

### 2.7. Reagents

- RNaseZap (Invitrogen by Thermo Fisher Scientific, Waltham, MA, USA; Cat. no.: AM9780).
- 10X TrypLE Select (Thermo Fisher Scientific–Gibco, Waltham, MA, USA; Cat. no.: A12177-01).
- Cell dissociation buffer (Thermo Fisher Scientific–Gibco, Waltham, MA, USA; Cat. no.: 13151-014).
- 1X sterile filtered Phosphate Buffered Saline (PBS) (Cytiva Hyclone, Marlborough, MA, USA; Cat. no.: SH30256.01).
- Bovine serum albumin (BSA) (Sigma-Aldrich, Singapore; Cat. no.: A9647-100G).
- Trypan blue stain 0.4% (Invitrogen by Thermo Fisher Scientific, Waltham,, USA; Cat. no.: T10282).
- DAPI (4′,6-diamidino-2-phenylindole) (Invitrogen by Thermo Fisher Scientific, Waltham, MA, USA; Cat. no.: D1306).

## 3. Procedure

### 3.1. Preparation of Materials and Reagents before Wing Dissection

(1) Collect pupae at the user-defined time point for the experiment. The number of wings required for the experiment depends on the experimental strategy (Appendix A).
(2) Clean all equipment and dissecting dishes with RNaseZap before starting the experiment.
(3) Gently wipe the outside of the pupae with 70% ethanol.
(4) Prepare 750 µL of 5X TrypLE (1:1 TrypLE:Cell dissocaiton buffer) for every three pupal wings being dissected. Keep it in an incubator at 37 °C.
(5) Prepare 1.5 mL Eppendorf tubes with 750 µL of 1X sterile filtered PBS.
(6) Precool the centrifuge to 4 °C.
(7) Prepare 10 mL of 1X sterile filtered PBS + 0.01% BSA and cool preparation by placing on ice.

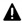
**.CRITICAL STEP** Prepare all reagents inside the Biological safely cabinet (BSC) to prevent bacterial contamination. If possible, perform the dissections within the BSC too.

### 3.2. Dissection of Wing Tissues

(1) Dissect pupal wing tissues at user-defined time points and place three wings in each Eppendorf tube with 750 µL of 1X sterile filtered PBS. Note B. anynana pupal forewings have an area of approximately 24 mm^2^. If using larger wings, perhaps place fewer wings per tube.
(2) Once dissections are done, gently pipette the wings in the existing 1X PBS to get rid of any attached pupal tissues or dirt.
(3) A detailed video protocol for the dissection of pupal wings in Bicyclus anynana is available [26].

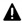
**.CRITICAL STEP** The developing butterfly wing disc is covered by a membrane called the periopidal membrane which contains many large cells. During pupal wing dissection, this membrane is clearly visible and can be removed very easily. For larval wings however, the peripodial membrane is very closely attached to the wing disc and can be removed after chemical treatments such as proteinase K. Without such pre-treatments, the membrane can be removed from larval wings by placing them in ice cold 1X PBS for 15–20 min before removal with a pair of very fine forceps. However, this is oftentimes inefficient in completely removing the peripodial membrane. Therefore, large peripodial membrane cells can potentially be sorted into the single-cell suspension of larval wing discs if sorting is based on size.

### 3.3. Cell Dissociation from the Wing Tissues

(1) Pipette out the 1X PBS and add 750 µL of warm 5X TrypLE to each tube containing the wings.
(2) Triturate with a 1000 µL pipette tip, by flushing the wings in and out of the pipette tip, until the wing discs are dissociated into smaller and finer pieces.
(3) Incubate the tubes in 37 °C for a total of 15–20 min while continuing the trituration step for around 30 s every 3–5 min. At the end of 15 min no wing tissue should be visible.

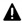
**.CRITICAL STEP** Triturate by moving the solution up and down the pipette tip gently but consistently every 3–5 min. This will help dissociate the cells and reduce the formation of clumps of cells.

(4) Top up each tube with 750 µL of 1X sterile filtered PBS to get a total volume of 1.5 mL.
(5) On top of a new 1.5 mL Eppendorf tube, place a 41 µm filter. Pass the 1.5 mL of cells through the filter by streaming the contents of the tube through it using a pipettor. Discard the filter.
(6) Centrifuge the filtered cells at 300 g, 4 °C for 5 min. You should see a very visible, slightly yellowish pellet at the bottom.

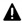
**.CRITICAL STEP** Keep the cells on ice at all times after this step to maintain high viability. Centrifuge at 4 °C and use ice cold buffers from this point onwards.

(7) Discard the supernatant without disturbing the pellet. Wash the pellet with 1 mL of ice cold 1X PBS + 0.01% BSA. Centrifuge again at 300 g, 4 °C for 5 min.
(8) Discard the supernatant and resuspend the pellet in 1mL of cold 1X PBS + 0.01% BSA using gentle pipetting. Tubes can be pooled at this stage. If pooling tubes, resuspend in required amount of cold 1X PBS + 0.01% BSA to get a final volume of 1 mL. Place on ice.
(9) Mix 5 µL of sample +5 µL of 0.4% trypan blue in a tube. Load 10 µL into the Countess cell counting chamber and count cells using the Countess Automated cell counter.

### 3.4. Fluorescence-Activated Cell Sorting

(1) Before going for the FACS, add 5 µL of DAPI (300 µM intermediate concentration) per 1 mL of filtered cells. Place on ice.
(2) To a 1.5 mL LoBind Eppendorf tube, add 500 µL of cold 1X PBS + 0.01% BSA and give a quick wash by shaking the tube and then pouring out the fluid. Add 50 µL of cold 1X PBS + 0.01% BSA and keep it on ice. This tube will be used to collect the sorted cells.
(3) Take the filtered cells and the LoBind collection tube on ice to the FACS facility.
(4) Perform an initial sorting using 3 µm, 6 µm, and 10 µm diameter reference beads and save the file as a reference.
(5) Sort the pupal wing cells based on the size profile using the beads as reference. We selected cells larger than 6 µm in diameter because we believe that these are the scale-building and socket cells at 24 h [10]. Other parameters are also used while sorting the cells such as sorting only single cells and sorting for live cells based on a DAPI stain.

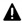
**.CRITICAL STEP** The large scale-building and socket cells (>6 µm) comprised about 7–8% of the total wing cell population in our experiment. Most cells in the wing tissue are around 3 µm in diameter which we believe are the underlying wing epidermal cells.

(6) Aim to collect about 100,000 cells with about 85% viability. Once the sorting is complete, place the collection tube on ice. We usually have about 500 µL after sorting.

### 3.5. Concentration of the Single-Cell Suspension

(1) In the lab, centrifuge the 500 µL of sorted cells at 900g, 4 °C for 3 min. A small pellet should be visible at the bottom.
(2) Remove the supernatant carefully without disturbing the pellet and discard.
(3) Resuspend the pellet in the required amount of 1X PBS + 0.01% BSA. This is determined by the user-specific requirments and experimental strategy (Appendix A).
(4) Estimate cell number per µL in your suspension before proceeding to sequencing library preparation. Since this sample is valuable, mix only 2.5 µL of the sample with 2.5 µL of 1X PBS + 0.01% BSA in a tube. To this add 5 µL of 0.4% trypan blue. Load the whole 10 µL volume into a Countess cell counting chamber and count the cells using the Countess Automated cell counter.

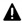
**.CRITICAL STEP** We used 10× Genomics for the following single cell partioning and library preparation. Depending on the experimental strategy, the 10× Genomics manual provides a tabular chart of required volumes of cell suspension given a stock concentration and required target cell recovery. In general, a minimum of 700–1200 cells/µL and >70% viability is required for the downstream experiments.

## 4. Expected Results

### 4.1. Dissection and Dissocation of Cells from the Wing Tissues

**Figure 3:**
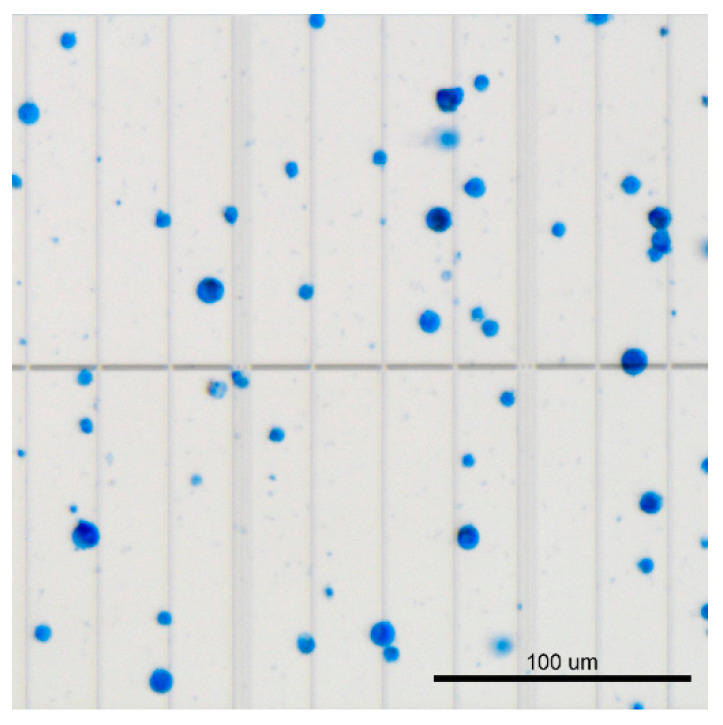
Trypan-blue-stained dissociated wing cells of 24-h-old pupal wings of *Bicyclus anynana* butterflies. The larger cells are probably scale-building and socket cells whereas the smaller cells are epidermal cells and other cell types.

*B. anynana* pupal wings at 24 h AP are fragile, translucent membranes with well defined trachae [26]. After cell dissocation from wing tissues using TrypLE, filtered cells stained with 0.4% trypan blue appear as a mixed population of different sized spherical cells (Figure 3). We usually get 2–4 million cells/mL upon pooling six to eight wings.

### 4.2. Fluorescence-Activated Cell Sorting

**Figure 4:**
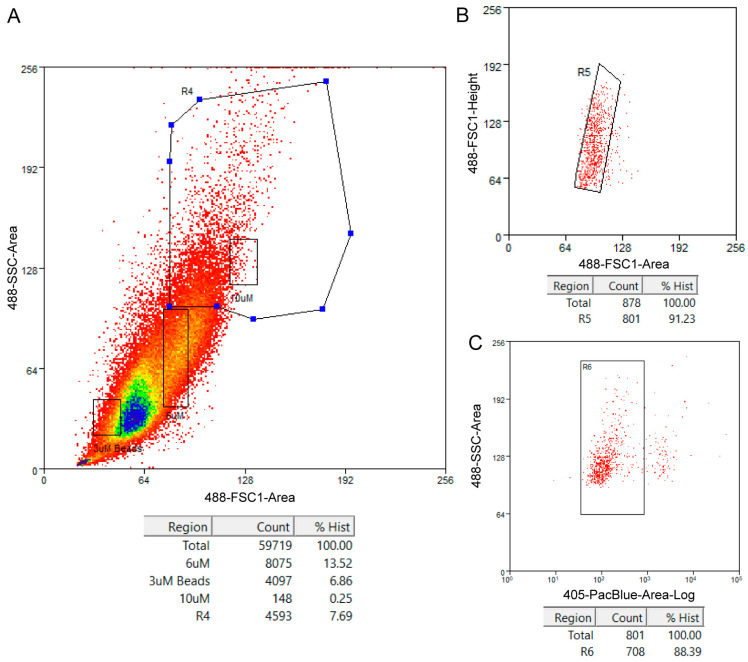
Cell population profiles of *Bicyclus anynana* 24 h pupal forewing cells indicating the gatings used in the fluorescence-activated cell sorting (FACS) procedure and associated statistics. (**A**) Side scatter area versus forward scatter area plot used to select cells based on size and granularity. Reference sizes are indicated by rectangles and the larger polygon R4 is the gating used to select desired cells. (**B**) Forward scatter height versus forward scatter area plot used to select for single cells and exclude any potential doublets in the cell suspension. The box R5 indicates selected cells. (**C**) Side scatter area versus fluorescence plot to select live cells. The box R6 indicates selected live cells based on lower DAPI fluorescence. The statistics inform about the percentage of cells captured via the gatings with respect to the total count.

The size distribution of the cells in the 24 h pupal wings of *B. anynana* is shown in Figure 4A. R4 is the gating we used to sort cells larger than 6 µm which we believe contain the scale-building cells and socket cells. Other parameters we used selected for single cells (Figure 4B) and for live cells (Figure 4C). For selecting live cells, we used DAPI, which is a cell-impermeant dye i.e., it is excluded from viable cells but produces a strong fluorescence upon binding to DNA in dead cells.

### 4.3. Concentration of Single-Cell Suspension

**Figure 5:**
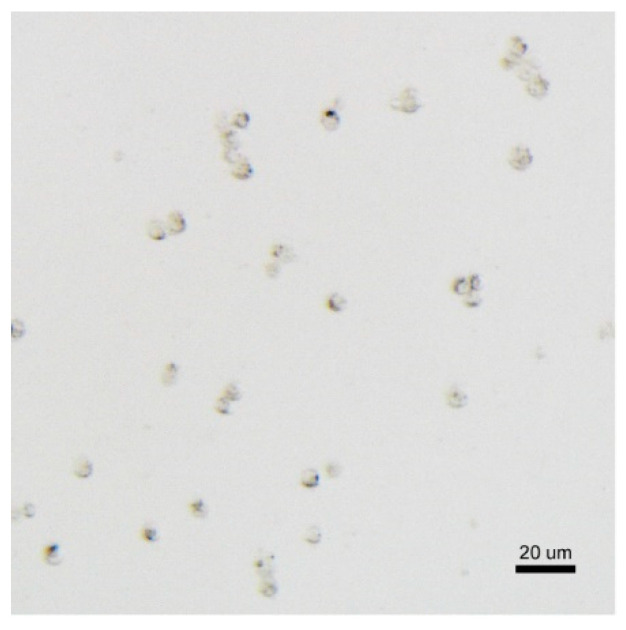
Unstained, dissociated, and FACS-sorted single-cell suspension of 24-h-old pupal wings of *Bicyclus anynana* butterflies.

After sorting and concentrating the single-cell suspension, a more uniform size distribution of cells is expected, with only the larger sorted cells present (Figure 5). A high concentration of live cells is required for downstream single-cell sorting and library preparation. In our case we used 10x Genomics.

## 5. Discussion

In this protocol, we have outlined, in detail, the steps to prepare a viable single-cell suspension from *B. anynana* 24 h pupal wing discs. This includes a FACS sorting step based on cell size, to specifically collect putative scale-building and socket cells which was our primary interest. However, there remain a number of challenges in using the technique of single-cell RNA sequencing to study scale development, especially with regard to the development of particular colors or patterns. Firstly, we used FACS to sort the single-cell wing suspension based on size. We selected for cells >6 µm to increase our chances of picking up putative scale-building and socket cells for the single-cell RNA sequencing and eliminating the much smaller wing epithelial cells. However, the wing tissue also contains larger cells such as hemolymph cells [27] that might be picked up in addition to the scale and socket cells. To overcome this, future studies can incorporate known antibody markers against SOP cells, such as the achaete-scute homolog (ASH) [23], during the FACS sorting step to pick out the scale precursors with certainty. Since these antibodies do not exist at present for butterflies, we did not use them in our study.

Secondly, the size of the scale-building cells varies across the wing, with larger cells generally producing longer scales such as the long scales that exist at the wing margin in many butterflies [25]. Sorting cells based on size could therefore exclude smaller scale-building and socket cells. This could again possibly be overcome by using antibodies to select specifically for scale cell precursors. Finally, cell dissociation from a bulk tissue leads to a loss of spatial information. At present, we would thus be unable to determine from which part of the wing disc a particular cell came. To overcome this, for example in the context of studying scale development within certain color pattern elements, antibodies against known marker genes for these pattern elements could be used in conjunction with SOP markers during the FACS sorting step. This would however be limited by the availability of markers for the different color pattern elements.

In summary, the protocol detailed in this manuscript for the preparation of a viable single-cell suspension from butterfly wing discs is vital for a successful single-cell experiment and for a more in-depth analysis of scale development and color pattern formation.

## Appendix A

Before the start of the experiment, the user is advised to outline an experimental strategy that determines the number of targeted single cells to be sequenced. We used 10x Genomics Chromium Single Cell 3′ (v3 Chemistry) [28] to partition the single cells into individual emulsion drops with barcoded beads followed by library preparation. This technique helps to profile a large number of cells at lower costs per cell but provides lower depth of profiling as compared to plate-based techniques such as Smart-seq2 [21,22]. The Chromium Single Cell 3′ (v3 Chemistry) User Guide (https://support.10xgenomics.com/single-cell-gene-expression/library-prep/doc/user-guide-chromium-single-cell-3-reagent-kits-user-guide-v3-chemistry) provides a detailed tabulation of how much of the prepared single cell suspension is required given a stock cell concentration and the expected target cell recovery. Increasing cell loading will increase the number of targeted cells in the experiment but will also increase multiplet rate i.e., the rate that two or more cells will be captured together in a droplet and will share the same barcoded bead. In our experiment, we pooled 14 male pupal forewing tissues between 22.5–25 h AP to obtain a good cell concentration with >70% viability, taking into consideration the FACS step used to sort out putative scale-building and socket cells. A smaller number of wings will be required if users wish to target fewer cells to be sequenced, if they do not wish to perform FACS, or have more experience with the protocol. We sorted 104,000 cells into 500 µL of 1X PBS + 0.01% BSA and resuspended the sorted cells in 30 µL of cold 1X PBS + 0.01% BSA. Our final cell count was 1650 cells/µL with 88% viability. Resuspension volumes can be smaller if increased cell concentrations are required in a particular experimental run.
